# 3-[(*E*)-3-(4-Meth­oxy­phen­yl)prop-2-eno­yl]-1-(4-methyl­phen­yl)-5-phenyl-1*H*-pyrazole-4-carbonitrile

**DOI:** 10.1107/S1600536811005770

**Published:** 2011-02-23

**Authors:** Hatem A. Abdel-Aziz, Ahmed Bari, Seik Weng Ng

**Affiliations:** a Department of Pharmaceutical Chemistry, College of Pharmacy, King Saud University, Riyadh 11451, Saudi Arabia; b Department of Chemistry, University of Malaya, 50603 Kuala Lumpur, Malaysia

## Abstract

In the title compound, C_27_H_21_N_3_O_2_, the non-H atoms of the meth­oxy­phenyl­acryloyl substitutent of the pyrazolyl ring are almost co-planar (r.m.s. deviation = 0.070 Å), and the mean plane is twisted by 18.7 (1)° with respect to the pyrazolyl ring. The phenyl and tolyl substituents are aligned at 48.9 (1) and 44.5 (1)° with respect to the pyrazolyl ring. Weak inter­molecular C—H⋯O and C—H⋯N hydrogen bonding is present in the crystal structure.

## Related literature

For background to the biological properties of aryl-substituted pyrazoles, see: Abdel-Aziz *et al.* (2010 ▶, 2011 ▶).
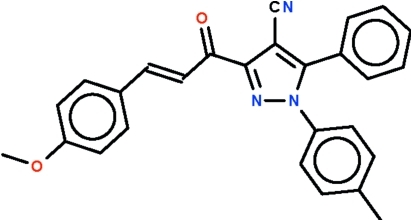


## Experimental

### 

#### Crystal data

C_27_H_21_N_3_O_2_
*M* *_r_* = 419.47
Triclinic, 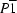

*a* = 10.9995 (7) Å
*b* = 11.0531 (8) Å
*c* = 11.4381 (8) Å
α = 95.113 (6)°
β = 111.582 (6)°
γ = 118.219 (7)°
*V* = 1079.13 (18) Å^3^
*Z* = 2
Mo *K*α radiation
μ = 0.08 mm^−1^
*T* = 100 K
0.20 × 0.15 × 0.05 mm

#### Data collection

Agilent SuperNova Dual diffractometer with Atlas detector
Absorption correction: multi-scan (*CrysAlis PRO*; Agilent, 2010 ▶) *T*_min_ = 0.984, *T*_max_ = 0.996
8250 measured reflections
4779 independent reflections
3346 reflections with *I* > 2σ(*I*)
*R*_int_ = 0.033

#### Refinement

*R*[*F* ^2^ > 2σ(*F* ^2^)] = 0.053
*wR*(*F* ^2^) = 0.133
*S* = 1.05
4779 reflections
291 parameters
H-atom parameters constrained
Δρ_max_ = 0.21 e Å^−3^
Δρ_min_ = −0.22 e Å^−3^

### 

Data collection: *CrysAlis PRO* (Agilent, 2010 ▶); cell refinement: *CrysAlis PRO*; data reduction: *CrysAlis PRO*; program(s) used to solve structure: *SHELXS97* (Sheldrick, 2008 ▶); program(s) used to refine structure: *SHELXL97* (Sheldrick, 2008 ▶); molecular graphics: *X-SEED* (Barbour, 2001 ▶); software used to prepare material for publication: *publCIF* (Westrip, 2010 ▶).

## Supplementary Material

Crystal structure: contains datablocks global, I. DOI: 10.1107/S1600536811005770/xu5162sup1.cif

Structure factors: contains datablocks I. DOI: 10.1107/S1600536811005770/xu5162Isup2.hkl

Additional supplementary materials:  crystallographic information; 3D view; checkCIF report

## Figures and Tables

**Table 1:** Hydrogen-bond geometry (Å, °)

*D*—H⋯*A*	*D*—H	H⋯*A*	*D*⋯*A*	*D*—H⋯*A*
C12—H12⋯O1^i^	0.95	2.59	3.350 (3)	137
C22—H22⋯N3^ii^	0.95	2.61	3.487 (3)	154
C25—H25⋯O2^iii^	0.95	2.56	3.484 (3)	164

Symmetry codes: (i) 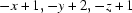; (ii) 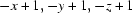; (iii) 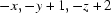.

**Fig. 1.**
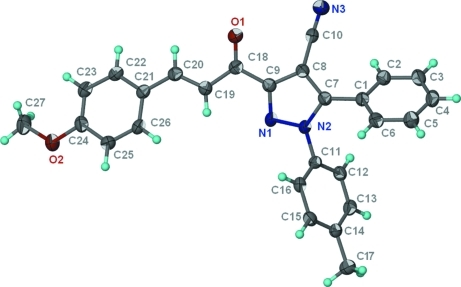
Thermal ellipsoid plot (Barbour, 2001) of C27H21N3O2 at the 70% probability level; hydrogen atoms are drawn as spheres of arbitrary radius.

## supplementary crystallographic information

### Comment

We have reported the antitumor activity of aryl-pyrazoles against CaCo-2 and HEP-2 cell lines (Abdel-Aziz *et al.*, 2010). Among these is the title compound (Scheme I), whose biological properties will be reported elsewhere (Abdel-Aziz *et al.*, 2011). The compound has methoxyphenylacryloyl, phenyl and tolyl substituents in the pyrazolyl ring. The methoxyphenylacryloyl substituent is twisted by 18.7 (1)° with respect to the pyrazolyl ring; the phenyl and tolyl substituents are aligned at 48.9 (1)° and 44.5 (1)° with respect to the five-membered ring (Fig. 1).

### Experimental

The synthesis will be reported elsewhere (Abdel-Aziz *et al.*, 2011). 3-Acetyl-5-phenyl-1-*p*-tolyl-1*H*-pyrazole-4-carbonitrile (10 mmol) was reacted with 4-methoxybenzaldehyde (10 mmol) in presence of sodium ethoxide solution (prepared by dissolving 0.23 g sodium metal in 50 ml absolute ethanol). The compound was recrystallized from an ethanol-DMF (3:1) mixture.

### Refinement

Carbon-bound H-atoms were placed in calculated positions [C—H 0.95 to 0.98 Å, *U*_iso_(H) 1.2 to 1.5*U*_eq_(C)] and were included in the refinement in the riding model approximation.

### Crystal data

**Table:**

C_27_H_21_N_3_O_2_	*Z* = 2
*M**_r_* = 419.47	*F*(000) = 440
Triclinic, *P*1	*D*_x_ = 1.291 Mg m^−^^3^
Hall symbol: -P 1	Mo *K*α radiation, λ = 0.71073 Å
*a* = 10.9995 (7) Å	Cell parameters from 2764 reflections
*b* = 11.0531 (8) Å	θ = 2.2–29.3°
*c* = 11.4381 (8) Å	µ = 0.08 mm^−^^1^
α = 95.113 (6)°	*T* = 100 K
β = 111.582 (6)°	Prism, colorless
γ = 118.219 (7)°	0.20 × 0.15 × 0.05 mm
*V* = 1079.13 (18) Å^3^	

### Data collection

**Table:**

Agilent SuperNova Dual diffractometer with Atlas detector	4779 independent reflections
Radiation source: SuperNova (Mo) X-ray Source	3346 reflections with *I* > 2σ(*I*)
Mirror	*R*_int_ = 0.033
Detector resolution: 10.4041 pixels mm^-1^	θ_max_ = 27.5°, θ_min_ = 2.2°
ω scan	*h* = −10→13
Absorption correction: multi-scan (*CrysAlis PRO*; Agilent, 2010)	*k* = −13→14
*T*_min_ = 0.984, *T*_max_ = 0.996	*l* = −14→14
8250 measured reflections	

### Refinement

**Table:**

Refinement on *F*^2^	Primary atom site location: structure-invariant direct methods
Least-squares matrix: full	Secondary atom site location: difference Fourier map
*R*[*F*^2^ > 2σ(*F*^2^)] = 0.053	Hydrogen site location: inferred from neighbouring sites
*wR*(*F*^2^) = 0.133	H-atom parameters constrained
*S* = 1.05	*w* = 1/[σ^2^(*F*_o_^2^) + (0.0431*P*)^2^ + 0.3112*P*] where *P* = (*F*_o_^2^ + 2*F*_c_^2^)/3
4779 reflections	(Δ/σ)_max_ = 0.001
291 parameters	Δρ_max_ = 0.21 e Å^−^^3^
0 restraints	Δρ_min_ = −0.22 e Å^−^^3^

### Fractional atomic coordinates and isotropic or equivalent isotropic displacement parameters (Å^2^)

**Table:**

	*x*	*y*	*z*	*U*_iso_*/*U*_eq_	
O1	0.38684 (16)	0.57825 (15)	0.44798 (13)	0.0300 (3)	
O2	0.12051 (18)	0.40793 (16)	1.05036 (14)	0.0367 (4)	
N1	0.23698 (18)	0.80105 (17)	0.43686 (15)	0.0234 (4)	
N2	0.24257 (18)	0.88927 (17)	0.36089 (15)	0.0224 (4)	
N3	0.5147 (2)	0.73362 (19)	0.22677 (18)	0.0346 (4)	
C1	0.3312 (2)	0.9656 (2)	0.19190 (18)	0.0240 (4)	
C2	0.4740 (2)	1.0450 (2)	0.1918 (2)	0.0279 (4)	
H2	0.5633	1.0516	0.2562	0.033*	
C3	0.4859 (3)	1.1146 (2)	0.0974 (2)	0.0333 (5)	
H3	0.5835	1.1689	0.0975	0.040*	
C4	0.3566 (3)	1.1051 (2)	0.0037 (2)	0.0345 (5)	
H4	0.3654	1.1535	−0.0602	0.041*	
C5	0.2142 (3)	1.0253 (2)	0.0027 (2)	0.0351 (5)	
H5	0.1252	1.0183	−0.0626	0.042*	
C6	0.2004 (2)	0.9555 (2)	0.09631 (19)	0.0296 (5)	
H6	0.1024	0.9011	0.0954	0.036*	
C7	0.3174 (2)	0.8880 (2)	0.28932 (18)	0.0232 (4)	
C8	0.3647 (2)	0.7940 (2)	0.32243 (18)	0.0231 (4)	
C9	0.3105 (2)	0.7419 (2)	0.41342 (18)	0.0230 (4)	
C10	0.4468 (2)	0.7579 (2)	0.26995 (19)	0.0253 (4)	
C11	0.1709 (2)	0.9692 (2)	0.36361 (17)	0.0219 (4)	
C12	0.2558 (2)	1.1174 (2)	0.39088 (18)	0.0242 (4)	
H12	0.3621	1.1678	0.4095	0.029*	
C13	0.1838 (2)	1.1919 (2)	0.39070 (18)	0.0258 (4)	
H13	0.2418	1.2942	0.4103	0.031*	
C14	0.0275 (2)	1.1190 (2)	0.36219 (18)	0.0237 (4)	
C15	−0.0541 (2)	0.9696 (2)	0.33824 (18)	0.0254 (4)	
H15	−0.1599	0.9188	0.3211	0.031*	
C16	0.0174 (2)	0.8946 (2)	0.33918 (17)	0.0235 (4)	
H16	−0.0386	0.7929	0.3232	0.028*	
C17	−0.0532 (3)	1.1984 (2)	0.3558 (2)	0.0328 (5)	
H17A	0.0250	1.3027	0.3984	0.049*	
H17B	−0.1163	1.1647	0.4020	0.049*	
H17C	−0.1209	1.1796	0.2627	0.049*	
C18	0.3238 (2)	0.6344 (2)	0.47637 (18)	0.0238 (4)	
C19	0.2638 (2)	0.6043 (2)	0.57209 (19)	0.0258 (4)	
H19	0.1994	0.6368	0.5774	0.031*	
C20	0.2979 (2)	0.5315 (2)	0.65250 (18)	0.0250 (4)	
H20	0.3597	0.4983	0.6416	0.030*	
C21	0.2506 (2)	0.4980 (2)	0.75476 (18)	0.0235 (4)	
C22	0.3173 (2)	0.4435 (2)	0.84454 (19)	0.0259 (4)	
H22	0.3919	0.4272	0.8368	0.031*	
C23	0.2780 (2)	0.4123 (2)	0.94527 (19)	0.0274 (4)	
H23	0.3254	0.3755	1.0058	0.033*	
C24	0.1695 (2)	0.4353 (2)	0.95637 (19)	0.0285 (5)	
C25	0.0980 (2)	0.4876 (2)	0.8662 (2)	0.0309 (5)	
H25	0.0217	0.5016	0.8729	0.037*	
C26	0.1392 (2)	0.5187 (2)	0.7676 (2)	0.0280 (4)	
H26	0.0911	0.5550	0.7069	0.034*	
C27	0.1885 (3)	0.3532 (3)	1.1453 (2)	0.0384 (5)	
H27A	0.1401	0.3340	1.2040	0.058*	
H27B	0.3008	0.4251	1.1981	0.058*	
H27C	0.1710	0.2632	1.0988	0.058*	

### Atomic displacement parameters (Å^2^)

**Table:**

	*U*^11^	*U*^22^	*U*^33^	*U*^12^	*U*^13^	*U*^23^
O1	0.0359 (8)	0.0295 (8)	0.0345 (8)	0.0213 (7)	0.0202 (7)	0.0137 (7)
O2	0.0487 (10)	0.0439 (9)	0.0351 (8)	0.0303 (8)	0.0269 (8)	0.0228 (8)
N1	0.0264 (9)	0.0237 (8)	0.0233 (8)	0.0147 (8)	0.0126 (7)	0.0115 (7)
N2	0.0256 (9)	0.0241 (8)	0.0229 (8)	0.0154 (8)	0.0130 (7)	0.0111 (7)
N3	0.0394 (11)	0.0351 (10)	0.0423 (11)	0.0239 (9)	0.0255 (9)	0.0159 (9)
C1	0.0312 (11)	0.0218 (10)	0.0229 (10)	0.0155 (9)	0.0147 (9)	0.0080 (8)
C2	0.0316 (11)	0.0246 (10)	0.0310 (11)	0.0152 (9)	0.0178 (9)	0.0103 (9)
C3	0.0456 (13)	0.0264 (11)	0.0382 (12)	0.0187 (11)	0.0295 (11)	0.0137 (10)
C4	0.0575 (15)	0.0328 (12)	0.0295 (11)	0.0293 (12)	0.0278 (11)	0.0166 (10)
C5	0.0473 (14)	0.0437 (13)	0.0271 (11)	0.0306 (12)	0.0201 (10)	0.0170 (10)
C6	0.0328 (11)	0.0357 (12)	0.0274 (10)	0.0208 (10)	0.0170 (9)	0.0135 (10)
C7	0.0232 (10)	0.0252 (10)	0.0221 (9)	0.0132 (9)	0.0112 (8)	0.0080 (8)
C8	0.0227 (10)	0.0243 (10)	0.0236 (10)	0.0129 (9)	0.0116 (8)	0.0087 (8)
C9	0.0214 (10)	0.0238 (10)	0.0227 (10)	0.0118 (9)	0.0102 (8)	0.0070 (8)
C10	0.0283 (11)	0.0235 (10)	0.0289 (10)	0.0149 (9)	0.0159 (9)	0.0125 (9)
C11	0.0254 (10)	0.0271 (10)	0.0196 (9)	0.0179 (9)	0.0112 (8)	0.0099 (8)
C12	0.0213 (10)	0.0260 (10)	0.0255 (10)	0.0117 (9)	0.0122 (8)	0.0106 (9)
C13	0.0314 (11)	0.0242 (10)	0.0223 (10)	0.0155 (9)	0.0122 (9)	0.0089 (9)
C14	0.0302 (11)	0.0295 (11)	0.0186 (9)	0.0198 (9)	0.0127 (8)	0.0111 (8)
C15	0.0226 (10)	0.0335 (11)	0.0233 (10)	0.0160 (9)	0.0120 (8)	0.0123 (9)
C16	0.0252 (10)	0.0224 (10)	0.0218 (9)	0.0116 (9)	0.0113 (8)	0.0091 (8)
C17	0.0401 (13)	0.0365 (12)	0.0334 (11)	0.0266 (11)	0.0196 (10)	0.0147 (10)
C18	0.0215 (10)	0.0212 (10)	0.0251 (10)	0.0106 (9)	0.0093 (8)	0.0063 (8)
C19	0.0289 (11)	0.0245 (10)	0.0287 (10)	0.0156 (9)	0.0161 (9)	0.0108 (9)
C20	0.0250 (10)	0.0223 (10)	0.0267 (10)	0.0129 (9)	0.0114 (9)	0.0065 (9)
C21	0.0242 (10)	0.0197 (9)	0.0255 (10)	0.0120 (9)	0.0106 (8)	0.0072 (8)
C22	0.0254 (10)	0.0233 (10)	0.0291 (10)	0.0145 (9)	0.0111 (9)	0.0087 (9)
C23	0.0271 (11)	0.0253 (10)	0.0266 (10)	0.0145 (9)	0.0088 (9)	0.0098 (9)
C24	0.0341 (12)	0.0273 (11)	0.0258 (10)	0.0158 (10)	0.0164 (9)	0.0111 (9)
C25	0.0345 (12)	0.0343 (12)	0.0380 (12)	0.0236 (10)	0.0220 (10)	0.0181 (10)
C26	0.0334 (11)	0.0284 (11)	0.0308 (11)	0.0207 (10)	0.0164 (9)	0.0158 (9)
C27	0.0477 (14)	0.0395 (13)	0.0306 (11)	0.0243 (12)	0.0184 (11)	0.0185 (11)

### Geometric parameters (Å, °)

**Table:**

O1—C18	1.228 (2)	C13—C14	1.393 (3)
O2—C24	1.367 (2)	C13—H13	0.9500
O2—C27	1.432 (2)	C14—C15	1.394 (3)
N1—C9	1.335 (2)	C14—C17	1.505 (3)
N1—N2	1.358 (2)	C15—C16	1.386 (3)
N2—C7	1.361 (2)	C15—H15	0.9500
N2—C11	1.440 (2)	C16—H16	0.9500
N3—C10	1.146 (2)	C17—H17A	0.9800
C1—C2	1.391 (3)	C17—H17B	0.9800
C1—C6	1.396 (3)	C17—H17C	0.9800
C1—C7	1.474 (3)	C18—C19	1.461 (3)
C2—C3	1.390 (3)	C19—C20	1.340 (3)
C2—H2	0.9500	C19—H19	0.9500
C3—C4	1.378 (3)	C20—C21	1.456 (3)
C3—H3	0.9500	C20—H20	0.9500
C4—C5	1.382 (3)	C21—C22	1.390 (3)
C4—H4	0.9500	C21—C26	1.402 (3)
C5—C6	1.386 (3)	C22—C23	1.389 (3)
C5—H5	0.9500	C22—H22	0.9500
C6—H6	0.9500	C23—C24	1.378 (3)
C7—C8	1.389 (3)	C23—H23	0.9500
C8—C9	1.417 (3)	C24—C25	1.399 (3)
C8—C10	1.425 (3)	C25—C26	1.376 (3)
C9—C18	1.481 (3)	C25—H25	0.9500
C11—C12	1.379 (3)	C26—H26	0.9500
C11—C16	1.380 (3)	C27—H27A	0.9800
C12—C13	1.387 (3)	C27—H27B	0.9800
C12—H12	0.9500	C27—H27C	0.9800
			
C24—O2—C27	117.63 (16)	C16—C15—C14	120.69 (18)
C9—N1—N2	105.08 (14)	C16—C15—H15	119.7
N1—N2—C7	112.95 (14)	C14—C15—H15	119.7
N1—N2—C11	118.61 (14)	C11—C16—C15	119.32 (18)
C7—N2—C11	128.44 (15)	C11—C16—H16	120.3
C2—C1—C6	119.62 (18)	C15—C16—H16	120.3
C2—C1—C7	119.97 (17)	C14—C17—H17A	109.5
C6—C1—C7	120.36 (18)	C14—C17—H17B	109.5
C3—C2—C1	119.9 (2)	H17A—C17—H17B	109.5
C3—C2—H2	120.0	C14—C17—H17C	109.5
C1—C2—H2	120.0	H17A—C17—H17C	109.5
C4—C3—C2	120.2 (2)	H17B—C17—H17C	109.5
C4—C3—H3	119.9	O1—C18—C19	123.83 (18)
C2—C3—H3	119.9	O1—C18—C9	118.84 (17)
C5—C4—C3	120.02 (19)	C19—C18—C9	117.31 (16)
C5—C4—H4	120.0	C20—C19—C18	121.14 (18)
C3—C4—H4	120.0	C20—C19—H19	119.4
C4—C5—C6	120.5 (2)	C18—C19—H19	119.4
C4—C5—H5	119.8	C19—C20—C21	127.01 (18)
C6—C5—H5	119.8	C19—C20—H20	116.5
C5—C6—C1	119.7 (2)	C21—C20—H20	116.5
C5—C6—H6	120.1	C22—C21—C26	117.69 (18)
C1—C6—H6	120.1	C22—C21—C20	119.60 (17)
N2—C7—C8	105.58 (16)	C26—C21—C20	122.71 (17)
N2—C7—C1	124.52 (16)	C21—C22—C23	121.73 (18)
C8—C7—C1	129.79 (17)	C21—C22—H22	119.1
C7—C8—C9	105.63 (16)	C23—C22—H22	119.1
C7—C8—C10	125.37 (17)	C24—C23—C22	119.28 (18)
C9—C8—C10	128.98 (17)	C24—C23—H23	120.4
N1—C9—C8	110.75 (16)	C22—C23—H23	120.4
N1—C9—C18	120.93 (16)	O2—C24—C23	124.75 (18)
C8—C9—C18	128.29 (17)	O2—C24—C25	114.80 (18)
N3—C10—C8	177.4 (2)	C23—C24—C25	120.44 (18)
C12—C11—C16	121.29 (17)	C26—C25—C24	119.41 (19)
C12—C11—N2	119.98 (17)	C26—C25—H25	120.3
C16—C11—N2	118.73 (17)	C24—C25—H25	120.3
C11—C12—C13	119.05 (18)	C25—C26—C21	121.43 (18)
C11—C12—H12	120.5	C25—C26—H26	119.3
C13—C12—H12	120.5	C21—C26—H26	119.3
C12—C13—C14	120.95 (18)	O2—C27—H27A	109.5
C12—C13—H13	119.5	O2—C27—H27B	109.5
C14—C13—H13	119.5	H27A—C27—H27B	109.5
C13—C14—C15	118.65 (17)	O2—C27—H27C	109.5
C13—C14—C17	121.14 (18)	H27A—C27—H27C	109.5
C15—C14—C17	120.21 (18)	H27B—C27—H27C	109.5

### Hydrogen-bond geometry (Å, °)

**Table:**

*D*—H···*A*	*D*—H	H···*A*	*D*···*A*	*D*—H···*A*
C12—H12···O1^i^	0.95	2.59	3.350 (3)	137
C22—H22···N3^ii^	0.95	2.61	3.487 (3)	154
C25—H25···O2^iii^	0.95	2.56	3.484 (3)	164

Symmetry codes: (i) −*x*+1, −*y*+2, −*z*+1; (ii) −*x*+1, −*y*+1, −*z*+1; (iii) −*x*, −*y*+1, −*z*+2.
